# Supplementary material for: In Vivo Study of Inoculation Approaches and Pathogenicity in African Swine Fever
Source: Vet Sci. 2024 Sep 1;11(9):403. doi: 10.3390/vetsci11090403 (PMC11435879; doi:10.3390/vetsci11090403)
Supplement: Supplementary file 1 [file vetsci-11-00403-s001.zip › Supplementary Material S1-Real-time quantitative PCR (qPCR) procedure.pdf]

Supplementary Material S1: Real-time quantitative PCR (qPCR) procedure

1. Stock solutions

1.1 Nuclease-free sterile water and qPCR reaction master mix (2×conc.).

1.2 Primers are prepared at a concentration of 10 μmol/μl: forward primer sequence 5' -CTG-CTC-ATG-GTA-TCA-ATC-TTA-TCG-A-3'; reverse primer sequence 5' -GAT-ACC-ACA-AGA-TC(AG)-GCC-GT-3' .

1.3 Fluorescent-labelled hydrolysis probe is included at a concentration of 10 pmol/μl: 5'-FAM-CCA-CGG-GAG-GAA-TAC-CAA-CCC-AGT-G-3'- TAMRA.

2. qPCR amplification

2.1 Prepare the qPCR reaction mixture described below for each sample in a sterile 1.5 ml microcentrifuge tube. Prepare the master mix for the number of samples to be assayed but allowing for one extra sample.

Nuclease-free water (5 μl); (2× conc.) qPCR reaction master mix (10 μl); forward primer (0.4 μl); reverse primer (0.4 μl); fluorescent-labelled probe (0.4 μl).

2.2 Add 16.2 μl qPCR reaction mix to one well of an optical reaction plate for each sample to be assayed.

2.3 Add 3.8 μl of extracted DNA template or blank extraction control and securely cover each well with a cap.

2.4 Spin the plate for 1 minute in a suitable centrifuge to mix the contents of each well.

2.5 Place the plate in a CFX Connect Real-Time PCR Detection System (Bio-Rad Laboratories, Inc., China) with FAM fluorescence channel and run the following programme:

Temperature	Duration	Cycle Number
50 ℃	2 min	1
95 ℃	10 min	1
95 ℃	15 sec	40
58 ℃	1 min	
	+Plate Read	

2.6 Reading the results

The point where the fluorescence measurement is above the background signal and reaches the detectable level is called the cycle threshold (Ct), and this is determined automatically by the PCR equipment software. It will be the starting fluorescence point for considering a sample as positive.

In a positive sample, a sigmoid-shaped amplification curve will be obtained where the Ct value will be <40. Samples giving a Ct value ≥38 should be considered as doubtful if a sigmoidal plot is observed and the analysis should be repeated for confirmation. A negative sample will maintain the fluorescence profile under background fluorescence level and the equipment will not report any Ct value.
